# Papillary Squamotransitional Cell Carcinoma of the Uterine Cervix: A Case Report and Review of the Literature

**DOI:** 10.1155/2016/7107910

**Published:** 2016-08-30

**Authors:** Shomaila Aamir M. Akbar, Mutahir A. Tunio, Sadeq Al-Dandan, Kareema Mohammed Y. Salamah, Mushabbab AlAsiri

**Affiliations:** ^1^Radiation Oncology, Comprehensive Cancer Center, King Fahad Medical City, Riyadh 59046, Saudi Arabia; ^2^Surgical Pathology, Department of Pathology and Laboratory Medicine Administration, King Fahad Medical City, Riyadh 59046, Saudi Arabia; ^3^King Saud bin Abdulaziz University, Riyadh, Saudi Arabia; ^4^King Saud University, Riyadh, Saudi Arabia; ^5^Gynecology Oncology, Women's Hospital, King Fahad Medical City, Riyadh 59046, Saudi Arabia

## Abstract

*Introduction*. Papillary squamotransitional cell carcinoma (PSTCC) is an uncommon histopathological variant of squamous cell carcinoma (SCC) of the uterine cervix, which occurs in postmenopausal women.* Presentation of Case*. Herein, we describe a case of a 63-year-old woman who presented with 4-month history of postmenopausal vaginal bleeding. Vaginal examination revealed a fragile lesion of size 1 × 1 cm invading left posterior vaginal fornice and parametrium. Biopsy showed the presence of papillae containing fibrovascular cores lined by multilayered atypical epithelial cells resembling squamous and transitional cell epithelium, confirming the diagnosis of PSTCC of the uterine cervix. After staging work-up she was staged according to the International Federation of Gynecology and Obstetrics (FIGO) staging system 2009 as FIGO IIB, and she was started on extended field concurrent chemoradiation.* Discussion*. PSTCC of the uterine cervix is an extremely rare and aggressive entity. PSTCC is often characterized by the presence of papillary structures with prominent fibrovascular cores. PSTCC of the uterine cervix should be differentiated from transitional cell carcinoma, squamous papilloma, papillary adenocarcinoma, and cervical intraepithelial neoplasia with papillary features.* Conclusion*. PSTCC of the uterine cervix is a diagnostic challenge; further studies regarding the mechanism underlying the development of PSCC are warranted.

## 1. Introduction

Papillary squamotransitional cell carcinoma (PSTCC) of the uterine cervix is an uncommon histopathological variant [1]. PSTCC have been reported also at other sites of the female genital tract (uterus, ovaries, and vagina) [2, 3]. These tumors show a broad spectrum of morphology, as these can appear as either “papillary squamous cell carcinoma (PSCC)” or “papillary transitional cell carcinoma (PTCC)” or mixed (PSTCC) [4]. However, a large series have shown that PSTCC variant is more predominant (50%) as compared to PSCC (28.1%) and PTCC (21.9%) [5].

As PSTCC are typically recognized due to its distinctive pattern of papillary growth pattern, these tumors should be segregated from transitional cell carcinoma, squamous papilloma, verrucous carcinoma, papillary serous adenocarcinoma, and cervical intraepithelial neoplasia especially grade III with papillary features [6, 7].

PSTCC of the uterine cervix is considered to be associated with an aggressive biologic behavior, as it mainly presents at a more advanced stage, and high propensity for locoregional recurrence and distant metastasis, in spite of the histopathological evidence suggesting a superficial or early invasive lesion [8].

Herein we present the clinicopathological presentation of PSTCC of the uterine cervix in a 63-year-old postmenopausal woman and relevant literature review.

## 2. Case Presentation

A 63-year-old female presented in our clinic with 4-month history of postmenopausal bleeding and pelvic pain, which was increasing in intensity over two months; however, she denied any weight loss, fever, or altered bowel habits. Her previous medical history revealed hypertension and chronic left ventricular dysfunction since the last 10 years; for that she was taking antihypertensive drugs and warfarin 5 mg daily. On physical assessment, she was pale with good performance status (Eastern Co-operative Oncology Group; ECOG-1). Per vaginal examination showed a fragile easily bleeding fungating lesion of size 1 × 1 cm invading the left posterior vaginal fornice and left parametrium. Hematology showed low hemoglobin (8.1 grams/deciliter), while other laboratory tests including serum electrolytes, renal, and hepatic function tests were found within normal limits.

**Figure 1:**
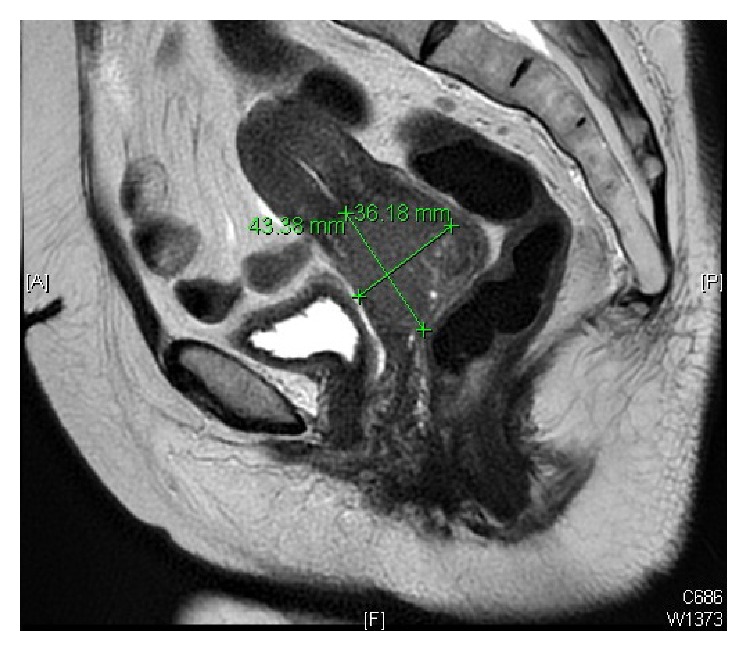
Sagittal view of pelvic MRI showing 4.3 × 3.6 cm cervical mass extending to the lower uterine segment (LUS) and left vaginal fornix with evidence of parametrial invasion on the left side.

**Figure 2:**
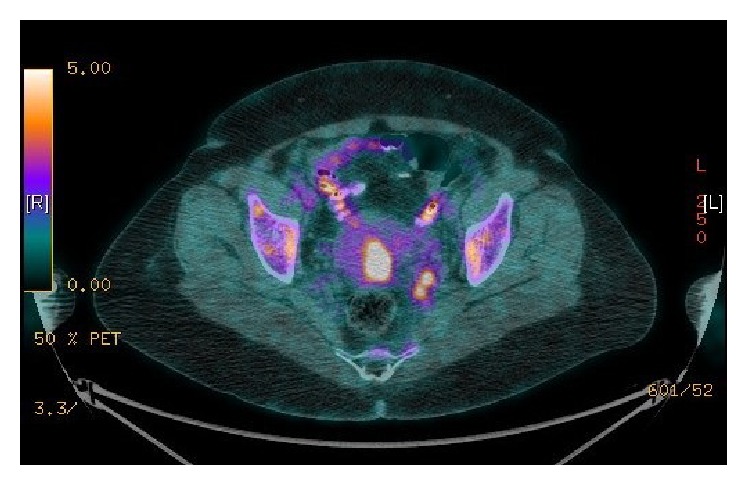
CT-PET imaging showing uptake in two left internal iliac lymph nodes and uptake in right external iliac small lymph node.

Magnetic resonance imaging (MRI) showed 4.3 × 3.6 cm cervical mass extending to the lower uterine segment (LUS) and left vaginal fornix with evidence of parametrial invasion on the left side and bilateral pelvic lymphadenopathy. Two left internal iliac enlarged lymph nodes, the largest measuring about 1.1 cm at short axis, and right external iliac small lymph node measuring 0.7 cm at short axis (Figure 1) were present. Computed tomography- (CT-) positron emission tomography (PET) confirmed the MRI findings with no evidence of distant metastasis (Figure 2).

**Figure 3:**
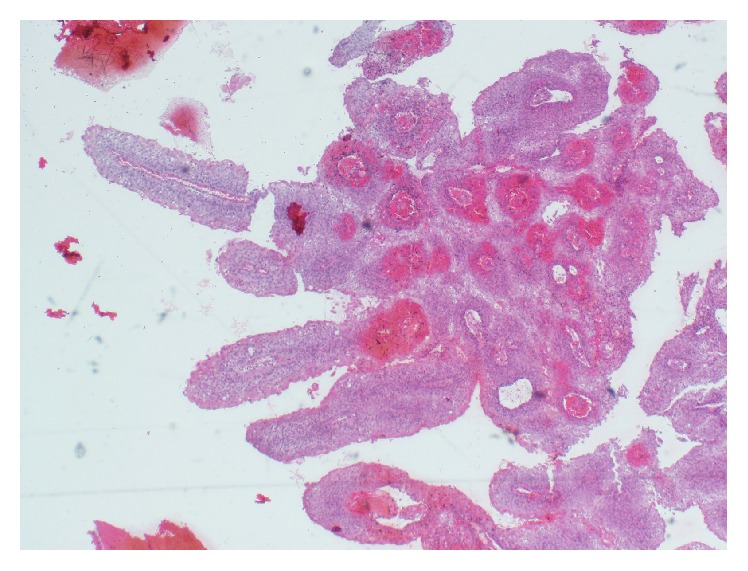
Cervical biopsy superficial fragment of papillary structures each containing a delicate fibrovascular core (H&E stain, 40x magnifications).

**Figure 4:**
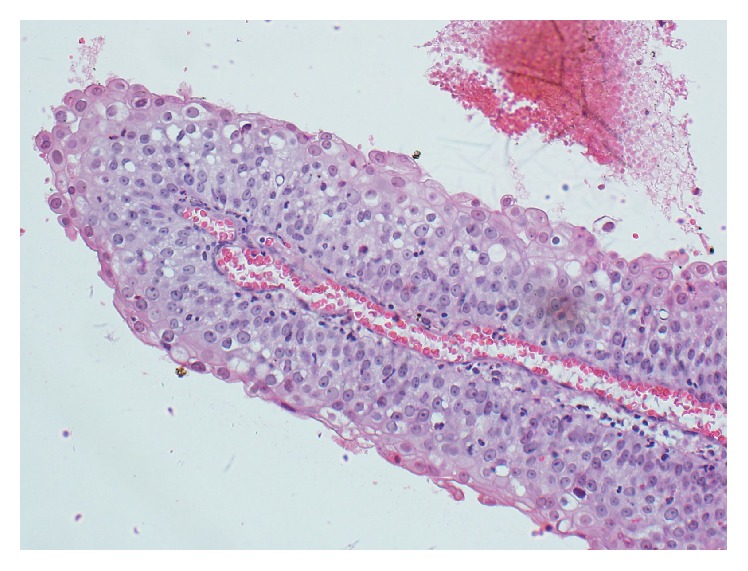
Papillary formation with squamotransitional cells having polygonal shape, distinct cell borders, abundant eosinophilic cytoplasm, pleomorphic nuclei, prominent nucleoli, and occasional mitotic figures (H&E stain, 200x magnifications).

A cervical biopsy was taken. Hematoxylin and eosin (H&E) staining showed a superficial fragment of papillary structures, each containing a delicate fibrovascular core, lined by multilayered atypical epithelial cells resembling squamous and transitional type epithelium, favoring the diagnosis of PSTCC (Figures 3 and 4).

After staging work-up she was staged according to the International Federation of Gynecology and Obstetrics (FIGO) staging system 2009 as FIGO IIB. After the multidisciplinary board meeting, patient was started on extended field concurrent chemoradiation. Radiotherapy dose was prescribed to para-aortic and pelvic fields at 45 Gy in 25 fractions at 1.8 Gy/fraction, followed by a parametrial boost (9 Gy at 1.8 Gy/5 fractions to complete 54 Gy) with concurrent weekly cisplatin, followed by high dose rate (HDR) brachytherapy of 21 Gy in 3 sessions.

## 3. Discussion

PSTCC of the uterine cervix is an extremely rare entity, and the presence of bland-looking basaloid cells or high grade squamous intraepithelial lesions (HSILs), together with scantiness of malignant cells, may lead to underdiagnosis of these histopathological variants [1, 9, 10]. Thus the identification of the subtle cytological characteristics with clinical correlation is mandatory to conclude diagnostic and staging dilemma. Further, on histopathological specimens, the stromal invasion is very difficult to see unless deep biopsies are taken, which can be a difficult process due to complex papillary architecture of PSTCC. However, in most of reported cases, stromal invasion varies between 55% and 65% [1, 5, 6].

The clinical manifestation of our patients was similar to other cases reports, that is, age above 60 years with postmenopausal bleeding [5–8]. However, our patient presented with severe vaginal bleeding, severe anemia, and presence of pelvic lymphadenopathy in spite of small cervical lesion.

Though follow-up period was short in our patient, PSTCC is known to be associated with delayed locoregional recurrences and metastasis [5, 11]. Tumor-suppressor protein p16 to predict the presence of human papillomavirus (HPV) was not investigated in our patients; however, previously published reports have supported the association of HPV and progression of PSTCC through an in situ phase [1, 5, 12].

In conclusion, PSTCC of uterine cervix is an uncommon histopathological variant, and the true prevalence remained undefined due to presence of bland-looking basaloid cells or HSILs, together with scantiness of malignant cells. PSTCC of uterine cervix is commonly seen in women aged above 60 years, known to be associated with HPV and present with delayed locoregional recurrences and distant metastasis. Due to rarity of PSTCC, treatment is similar to squamous cell carcinoma or adenocarcinoma of cervix.
